# Key Characteristics and Development of Psychoceuticals: A Review

**DOI:** 10.3390/ijms232415777

**Published:** 2022-12-12

**Authors:** Genaro Herrera Cano, Jordan Dean, Samuel Padilla Abreu, Amanda Hernández Rodríguez, Cyrena Abbasi, Madison Hinson, Brandon Lucke-Wold

**Affiliations:** 1 University of Connecticut School of Medicine, 263 Farmington Ave, Farmington, CT 06030, USA; 2 Wake Forest University School of Medicine, 475 Vine St, Winston-Salem, NC 27101, USA; 3 Department of Neurosurgery, University of Florida, Gainesville, FL 32608, USA

**Keywords:** psychoceutical, neurointervention, pharmacotherapy, emerging treatment, improved outcomes

## Abstract

Psychoceuticals have brought benefits to the pharmacotherapeutic management of central nervous system (CNS) illnesses since the 19th century. However, these drugs have potential side effects or lack high response rates. This review covers twenty drugs’ biochemical mechanisms, benefits, risks, and clinical trial reports. For this study, medications from seven psychoceutical organizations were reviewed and evaluated. Nineteen drugs were chosen from the organizations, and one was selected from the literature. The databases used for the search were Pubmed, Google Scholar, and NIH clinical trials. In addition, information from the organizations’ websites and other sources, such as news reports, were also used. From the list of drugs, the most common targets were serotonergic, opioid, and N-methyl-D-aspartate (NMDA) receptors. These drugs have shown promise in psychiatric illnesses such as substance abuse, post-traumatic stress disorder (PTSD), anxiety, depression, and neurological conditions, such as Parkinson’s disease, traumatic brain injury, and neuroinflammation. Some of these drugs, however, are still early in development, so their therapeutic significance cannot be determined. These twenty drugs have promising benefits, but their clinical usage and efficacy must still be explored.

## 1. Introduction

The neurological and psychiatric pharmacology fields have grown immensely since the 19th century and have brought considerable benefits to managing and treating various illnesses. For example, the introduction of morphine as well as other medications that induced sedation for the rapid control of agitation and aggression in psychiatric hospitals during the second half of the 19th century [1,2] sparked research into pharmacotherapeutic interventions for neurologic and mental health illnesses. By the late 1950s, the discovery of six different central nervous system (CNS) neurotransmitters (acetylcholine, dopamine, GABA, norepinephrine, serotonin, and substance P) allowed the development of pharmacotherapeutic treatments for neurological conditions, such as Parkinson’s disease, and psychiatric conditions, such as depression and schizophrenia [1,2]. In contrast, there appears to be less interest in the research and development of psychoceutical drugs within the 21st century.

The current state of psychoceutical innovation has remained motionless for a long time. Many of the drugs within this class used today were discovered more than 70 years ago [2,3]. Various factors surrounding the research and trial testing of psychoceuticals have kept the development of these drugs from moving forward. Some examples are limited pathophysiological knowledge [2,3], high rates of failure in phase III trials, poor predictive animal models [2], later returns of investment, and high costs of drug development [4,5], among others. These factors negatively impact the development of new medications in this class, yet the most important one is the shift of charge from clinical researchers to the pharmaceutical industry. Clinical researchers are restricted to supporting the pharmaceutical industry’s interests [1,6,7,8], which can increase the risk of bias. Such restriction of data to the pharmacotherapy community can potentially harm the patients these products treat. Therefore, it is crucial to monitor the development of novel psychoceuticals that aim to replace or improve pharmacotherapy in the global market.

Currently, the psychoceuticals available in the global market are accompanied by multiple side effects and have response rates of less than 50% [2]. To see a positive change in neurological and psychiatric pharmacotherapy, the clinical research community must support the moral development of novel drugs within those fields. The main focus of developing new modes of treatment should be to improve patient care management instead of supporting the pharmaceutical industry’s interests. Furthermore, the pathophysiology behind the illnesses these fields target must be further explored. A clear understanding of the biochemical mechanisms of neurological and psychiatric conditions is the key to the success of clinical drug production [4]. Although there are other issues to tackle in developing psychoceuticals, these two should be targeted first to improve neurological and psychiatric pharmacotherapy immensely.

Over the years, psychoceuticals have come a long way in medical research to explore their benefits, risks, and underlying biochemical mechanisms to achieve their outcomes. There is still room for testing regarding patient healthcare outcomes within neurological-related specialties. Currently, psychoceutical organizations are evaluating potential outcomes for old and newly engineered drugs. For this review, drug products from six different psychoceutical organizations were selected for evaluation. Nineteen drugs from the organizations and one more from the literature were selected to review for their biochemical pathway mechanisms, benefits and risks, and clinical trials.

## 2. Results and Discussion

**Figure 1:**
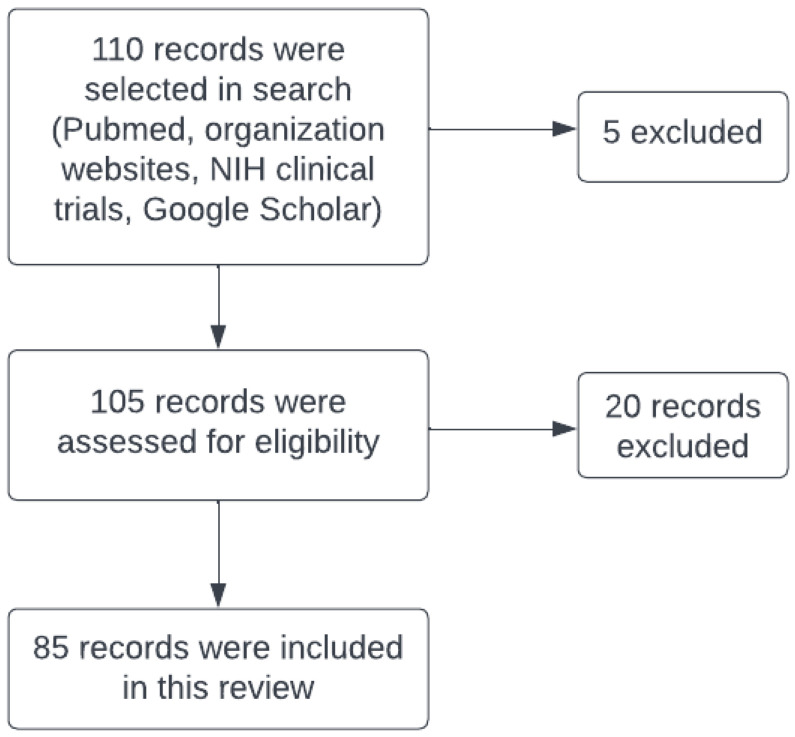
Flow Search Diagram.

A total of 110 records were selected in the search (Pubmed, NIH clinical trials, organization websites, and Google Scholar). Before assessing eligibility, five were excluded due to duplication of the records. Then, 20 records were excluded due to there being no published clinical trial studies or the results not being posted for initiated studies (Figure 1).

### 2.1. Biochemical Mechanisms of Drugs

The psychoceutical list is composed of drugs interacting with various CNS targets. Most of the drugs target serotonin receptors, followed by drugs that target NMDA and opioid receptors. Other targets include GLUT-8, NF-κB, α3β4 receptors, GABA receptors, and peptides (Table 1). It is essential to recognize that not all the biochemical pathways of these drugs are fully understood. Of the twenty drugs reviewed, nine target the 5-HT family of receptors (5-HT_1A_, 5-HT_2A_, 5-HT_1B_, 5-HT_2B_, 5-HT_2C_), two interact with opioid receptors (mu-opioid, k-opioid), and two interact with NMDA receptors. Two drugs target all three types of receptors. The other five drugs interact with one of the remaining targets. In general, serotonergic targeting drugs directly enhance the activity of these receptors or increase the serotonin levels in the synaptic cleft by blocking reuptake transporters. Opioid-targeting drugs increase the activity of the G-protein signaling and other pathways, such as the beta-arrestin and ERK/MAPK pathways. NMDA-targeting drugs act as antagonists.

#### 2.1.1. Serotonin Receptor-Targeted Drugs

Most of the psychoceuticals reviewed fall within this target category. These drugs generally bind to CNS G-protein receptors within the 5-HT family, such as 5-HT_1A_, 5-HT_2A_, and 5-HT_2B_, and act agonistically [9,13,14,15,17,18]. Additionally, two of these, ecstasy and CYB005 (phenethylamine derivative [20]), block the reuptake of serotonin, increasing the serotonin levels within the synaptic cleft [12,21,22]. These drugs aim to increase the activity of serotonergic systems within the CNS.

Although these drugs are expected only to stimulate serotonergic systems, LSD demonstrates the opposite. LSD has a dual effect on brain systems, such as the locus coeruleus (LC), raphe nuclei (RN), and the brain cortex [14]. LSD’s agonistic activity on 5-HT_1A_ receptors has inhibitory effects [14]. In contrast, the agonistic activity of 5-HT_2_ receptors has stimulatory effects [14]. No reports of the other eight drugs performing inhibitory or dual serotonergic activities have been found. The possibility of these drugs causing inhibitory or dual effects through multiple serotonin receptor interactions should not be disregarded.

The underlying mechanisms activated after drug–receptor interactions have still not been confirmed for all nine psychoceuticals. Research on Peyote activity has revealed that 5-HT_2_ receptors, especially 5-HT_2C_ receptors coupled with G-proteins, activate phospholipase C [15]. This activation increases the cytosolic levels of IP3, causing endoplasmic reticulum calcium release into the cytosol of postsynaptic neurons [15]. It may be assumed that drugs interacting with 5-HT_2_ receptors (LSD, CYB003, CYB004, Psilocybin [14,16,17,18]) initiate cellular mechanisms similar to Peyote, but cannot be guaranteed at this time. For example, LSD has demonstrated a strong selectivity for the beta-arrestin pathway through 5-HT_2B_ receptor activity [13]. At the same time, there is unbiased selectivity between the beta-arrestin and G-protein signaling pathways through interactions with 5-HT_1B_ receptors [13]. Although these drugs target similar receptors, their underlying biochemical pathways may differ and should be investigated in future studies.

Furthermore, CNS enzymes can denature some serotonergic systems targeting psychoceuticals and impede excess system activity, but it is not always the case. The inactivation of 5-MeO-DMT by monoamine oxidase A produces an active metabolite, bufotenine, that binds with a higher affinity to 5-HT_2A_ receptors than the parent drug [9,10]. In contrast, the endogenous hallucinogen N, N-dimethyltryptamine (DMT) can be rendered inactive by monoamine oxidase (MAO) [11]. The CNS’ capacity for drug neutralization must be considered to modulate serotonergic system activity.

Serotonergic transport blockers give another option to affect CNS serotonergic system activity indirectly. According to preclinical data, CYB005, a phenethylamine derivative, aims to inhibit serotonin transporters within the synaptic cleft [20,21,22]. On the other hand, MDMA-derivative ecstasy blocks the reuptake of serotonin and norepinephrine and enhances their release into the synaptic cleft [12]. Furthermore, chronic use of ecstasy has been shown to cause a decrease in the expression of serotonin transporters, resulting in less serotonin reuptake and recycling [12]. However, the current focus of serotonergic drug research and development is directly stimulating serotonergic system activity.

#### 2.1.2. NMDA Receptor-Targeted Drugs

The psychoceuticals under this category, ketamine and arketamine (an enantiomer of ketamine), induce antidepressant effects by inhibiting NMDA receptors [24,26]. Ketamine’s non-competitive antagonistic activity on these receptors results in glutamate surges stimulating secondary AMPA receptor (AMPAr) activity as well [26,27]. This secondary activation is necessary to achieve ketamine’s effects [25]. While the underlying molecular mechanism of arketamine is still unclear, previous studies have shown this medication to have more potent and longer-lasting antidepressant effects than ketamine and other enantiomer forms [24,25].

The role of AMPArs after exposure to arketamine should be explored. If the parental structure requires AMPAr activation potentiating pathways, such as mTORC1 and BDNF, for antidepressant effects to occur [27], then it calls into question what mechanisms arketamine induces for similar yet more potent effects to arise. Two clinical trials will be conducted using perampanel, an AMPAr antagonist, in patients with treatment-resistant major depressive disorder, hoping to confirm the role of AMPAr in arketamine’s effects [25].

#### 2.1.3. Opioid Receptor Targeted Drugs

The drugs targeting opioid receptors, deu-mitragynine (a major alkaloid component of kratom [28]) and salvinorin A (a non-alkaloidal component of Salvia divinorum [30]), regulate pathways initiated by G-proteins, while also stimulating other mechanisms, such as the beta-arrestin and ERK/MAPK pathways. Kratom, a mu-opioid receptor (MOR) agonist with low potency, has been demonstrated to emit ‘atypical’ opioid effects (no respiratory depression, emesis, or shortness of breath in animal models) through G-protein agonistic activity and limited recruitment of beta-arrestin after opioid receptor-dependent activation [28,29]. Studies with rodent models alternatively suggest mitragynine and its derivatives do not directly activate opioid receptors [29], which brings into question how the drug initiates the activity of MORs to deliver analgesic effects.

In contrast, salvinorin A stimulates analgesic effects by acting as a selective agonist of k-opioid receptors (KOPr) [30]. Through this receptor, adenylate cyclase is inhibited, beta-arrestin is mediated, and the ERK/MAPK pathways are activated [31]. Salvinorin A also has decreased dopaminergic activation in the dorsal and ventral striata [31], suggesting a reduced risk for addiction compared to other opioids, such as morphine. These drugs can potentially help treat individuals suffering from opioid addiction by decreasing the sense of reward from opioid consumption or reducing the risk of lethal side effects.

#### 2.1.4. Multi-Receptor Targeted Drugs

The two psychoceuticals, ibogaine and noribogaine, are perplexing, as both can interact with opioid, serotonergic, and glutamatergic systems [32,33,34,35]. These systems include kappa-opioid, mu-opioid, NMDA, and serotonin reuptake receptors [32,33,34,35]. Ibogaine, however, can interact with nicotinic and sigma-2 receptors [32,33], while noribogaine cannot [34]. The ability to interact with multiple receptor systems can be a clinical concern, as unintended effects may occur, such as bradycardia and long QT syndrome [46].

The anti-addictive effects of ibogaine and noribogaine appear to be governed by the activity of the k-opioid receptor [34]. Rat model studies demonstrated that ibogaine’s k-opioid agonistic activity decreased cocaine and morphine self-administration [32]. In addition, noribogaine’s administration to human opioid-dependent subjects exhibited a decrease in withdrawal symptoms [47], most likely due to k-opioid agonistic activity [35]. The exact underlying mechanism that achieves this for both drugs remains unclear [32,34,35,47]. Avidor-Reiss’ study presented k-opioid agonist’s effects on adenylyl cyclase (AC) activity [48]. Acute exposure to kappa opioid agonists can potentiate the inhibition of AC, while chronic exposure can lead to the sensitization of AC activity [48]. The drug’s exact biochemical mechanisms should be researched extensively before further conclusions can be made. Additionally, these two drugs do not solely rely on opioid agonism [34], although the roles of other receptors in the anti-addictive effects are not entirely understood [32]. Exploring their role is key to understanding the potential effects of ibogaine and noribogaine.

#### 2.1.5. Other Receptor Targeted Drugs

Five of the twenty psychoceuticals evaluated in this review have mechanisms that do not involve serotonergic, opioid, or glutamatergic systems. The mechanisms for three of these drugs, SLS-007 (peptidic drug), zolunicant HCl (MM-110), and deuterated etifoxine, require further investigation. SLS-007 targets alpha-synuclein’s NAcore (non-amyloid component core) and inhibits protein aggregation in Parkinson’s patients, showing therapeutic potential in mouse models [40,41]. The mechanism by which it inhibits protein aggregation has not currently been reported. It is suspected that SLS-007 acts as an intrabody binding to alpha-synuclein monomers to prevent their oligomerization and, consequently, stop the formation of neurotoxic fibrils [40,49]. Zolunicant HCl is known to be a potent inhibitor of nicotinic alpha-3-beta-4 receptors [44,50] and manages the regulation of dopamine levels during substance withdrawal [51]. The underlying mechanism of this drug has yet to be reported. Deuterated etifoxine aims to boost depressant effects through the positive allosteric stimulation of the GABA receptor’s β 2 and β 3 subunits [45]. In addition, increased mitochondrial TSPO promotes neurosteroid synthesis [45], although the significance of inducing these effects is still under study. More research on the mechanisms of these three drugs should be performed to form conclusions about their biochemical behavior.

Compared to the research on these three drugs, there is more knowledge on the biochemical pathways of the other two psychoceuticals: Trehalose (SLS-005) and N-acetylcysteine (NAC).

Trehalose enhances autophagy and lysosomal activation through an mTOR-independent pathway and nuclear translocation of transcription factor EB [37,38,39]. The light chain 3–II (LC3-II) increase in the cytosol indicates this enhancement [36,37,38,39]. Promoting the fusion of autophagosomes to lysosomes, LC3-II leads to the degradation of autophagosome contents [36,37,38,39]. However, some observations suggest that trehalose can inhibit autophagic flux as well [38]. Therefore, the dual behavior of trehalose should be investigated further.

Lastly, NAC has been hypothesized to exert cytoprotective effects in the CNS [43]. Often used to increase intracellular glutathione for paracetamol overdose [42], the drug’s anti-inflammatory properties may allow for the modulation of inflammatory mechanisms in other areas of the body. By suppressing nuclear factor kappa B (NF-κB) activity, the compound reduces levels of cytokines, such as tumor necrosis factor-alpha (TNF-α) and interleukins (IL-6 and IL-1B) [42]. Using the drug’s anti-inflammatory properties can potentially reduce brain damage from exerted neuroinflammation.

### 2.2. Pre-Clinical/Clinical Benefits and Risks of Drugs

Pre-clinical and clinical studies testing the 20 psychoceuticals have shown benefits related to substance abuse, PTSD, anxiety, depression, and other diseases (Table 2). Although promising therapeutic results have been indicated for these drugs, there are reports of potential adverse effects from some of them. The nature of these side effects include psychiatric (agitation, panic attacks), neurological (ataxia, sensory dissociation), gastrointestinal (nausea/vomiting, epigastric pain), cardiovascular (tachycardia, hypertension), endocrine (increased cortisol, prolactin), and systemic (chills, headaches) signs and symptoms. Studies on Zolunicant HCl, CYB005, CYB004, and CYB003 were not found in the literature search, so this information was obtained through the websites of their respective pharmaceutical companies.

As commonly used analgesics, such as opioids, have a high risk of being addictive, psychoceuticals that are en route to being approved may eventually replace opioids in clinical practice. For instance, Kratom exhibits analgesic effects by acting as an MOR agonist [28,71]. In addition, psychedelics have also been demonstrated to help reduce cravings for patients undergoing withdrawal from opioid use. For example, MM-110 (zolunicant HCl) and noribogaine are currently being investigated for their anti-craving properties with opioid withdrawal through the inhibition of nicotinic α3β4 receptors [44] or activation of k-opioid receptors [34,55,56]. Ibogaine also has anti-craving effects for opioid withdrawal by activating the k-opioid receptor. In addition, ibogaine also alleviates the withdrawal symptoms of certain recreational drugs, such as cocaine [34,46].

Another of the most significant benefits observed with these drugs is their use in psychological disorders. Various studies have demonstrated improvement in humans after being exposed to these drugs. Some of the medications that have been shown to decrease depression and anxiety are LSD [52], peyote [58], ketamine [67], arketamine [24,63], N, N-dimethyltryptamine [11], psilocybin [64], and deuterated etifoxine [59,60]. Administration of 5-methoxy-N, N-dimethyltryptamine (5-MeO-DMT) has reduced suicidal ideations, planning, and attempts [53,54]. Ecstasy has shown an attenuated amygdala response in humans who are shown negative stimuli and an extinction of fear memories in mice [65,66]. Lastly, salvinorin A has been shown to create feelings of calm and relaxation, leading to an improved mood [30,61]. In contrast, it had endocrine effects that significantly raised cortisol and prolactin levels [30].

Besides these compounds, new ones have been engineered by combining novel psycoceutical molecules with controllable drug delivery systems through a deuterated process for possible mental health treatments. Three drugs that researchers have been working on are CYB003 (for depressive disorder), CYB004 (for anxiety disorders), and CYB005 (for neuroinflammation). CYB003 is a deuterated psilocybin analog that has received FDA IND clearance to enter clinical development [16]. CYB004 is a deuterated DMT being used to conduct phase I in-human studies [18]. Its preclinical studies demonstrated that it could potentially treat anxiety disorders [18]. Finally, CYB005 is a phenethylamine derivative currently in preclinical development [55], which has the potential to treat neuroinflammation and psychiatric conditions [20].

Other drugs reviewed have shown to be beneficial in areas outside of psychological disorders. For example, SLS-007, a peptidic inhibitor being tested in a preclinical study, can stop the propagation and seeding of α-synuclein aggregates, which could help in Alzheimer’s disease, Parkinson’s disease, and Lewy body dementia [40,70]. SLS-005, a Trehalose injection, is currently used in a phase II clinical trial for Alzheimer’s patients to test its treatment safety and efficacy [72]. In addition, it has been shown that it protects against oxidative stress, increases levels of chaperone molecules, and enhances autophagy [62]. Animal studies have also shown that it is neuroprotective and efficacious in improving functional outcomes following a traumatic brain injury (TBI) [62]. Finally, N-acetylcysteine, a well-established drug known to be used in patients with COPD, cystic fibrosis, or those with a Tylenol overdose, has been theorized to show beneficial effects in patients with schizophrenia by improving cognition [68,69].

Even though these compounds have shown promise pre-clinically and clinically, their usage can have potential side effects which are important to be aware of. These side effects can vary from mental state/consciousness alterations to physiological changes. The following are the general risks of consumption of these compounds:

- LSD includes delusions, visual hallucinations, distortion of one’s sense of time and identity, impaired depth and time perception, artificial sense of euphoria or certainty, distorted perception of the size and shape of objects, movements, colors, sounds, touch, and the user’s body image, severe, terrifying thoughts and feelings, fear of losing control, fear of death, and panic attacks [52].
- Reports of 5-MeO-DMT show agitation and tachycardia with periodic reports of hyperthermia, seizures, coma, increased serum creatinine, and life-threatening experiences, such as cardiac arrest and possible death [53].
- Ibogaine has side effects such as nausea, headache, and visual changes; the most common side effects are reported in [46].
- Noribogaine’s most common adverse events are headache and epistaxis [56].
- Peyote is classified as possessing the highest potential for use disorder and misuse [73].
- Salvinorin A has adverse outcomes, including fear, panic, paranoia, agitated delirium, sadness, irritability, augmented perspiration, and chills [30]. In addition, consumers can feel a lack of insight, making them susceptible to harm, which can occur during the performance of complex tasks, such as driving [30]. Gastrointestinal malaises (e.g., nausea and vomiting) have also been reported [30].
- Arketamine’s side effects include a slight rise in blood pressure, with a mean increase of 16.7 mmHg for systolic BP and 11.9 mmHg for diastolic [63]. Other adverse effects are ataxia, sensory dissociation, hyperactivity, and conditioned-place preference [24].
- Ecstasy’s reported side effects are heat strokes, relapsing of pre-existing schizophrenia [65], and depression [65,74]. This drug also has cardiovascular side effects, such as cardiac contractile dysfunction and vasoconstriction [75].
- Oral N-acetylcysteine (NAC) may cause nausea, vomiting, diarrhea, flatus, and gastroesophageal reflux [69]. In addition, IV NAC can cause rate-related anaphylactoid reactions [69].
- Ketamine’s side effects include nausea/vomiting and epigastric pain. A rapid ketamine IV injection can cause transient apnea, cystitis, and contracted bladder [67].
- N, N-dimethyltryptamine’s side effects include increased levels of corticotropin, cortisol, prolactin, and growth hormone when administered to human volunteers [11].
- Kratom has shown mental health effects, primarily withdrawal symptoms [71].

### 2.3. Clinical Trials

The chosen psychoceuticals to review are in different stages of development (pre-clinical trial, phase I clinical trial, phase II clinical trial). Therefore, the information available from each one can vary depending on how far in development they are. Of the 20 drugs, Kratom, SLS-007, N, N-dimethyltryptamine, Zolunicant HCl, CYB005, CYB004, CYB003, deuterated etifoxine, and SLS-005 do not currently have completed clinical trials.

Five of the eleven drugs have been tested for therapeutic effects in psychiatric-related illnesses. LSD has been shown to have anxiolytic, antidepressant, and anti-addictive properties [76]. However, LSD’s clinical research during the 1960s and 1970s was halted due to the rise in its recreational use [76]. A recent phase-II clinical trial reported some improvement in anxiety for individuals with advanced-stage life-threatening disease in the full-dose LSD group [77]. Similarly, there have been self-reports of 5-MeO-DMT improving anxiety and depression along with no changes in memory, attention, and cognitive function [78,79]. In treatment-resistant depression, arketamine and psilocybin significantly decreased depressive symptoms [63,80]. Adverse effects of headache, nausea, and dizziness after psilocybin treatment were reported [80]. Besides anxiety and depression, PTSD treatment has also been under evaluation. Ecstasy has been reported beneficial to the treatment of PTSD in a randomized phase-III clinical trial with significant improvements in CAPS-5 scores in the ecstasy group compared to the control group, along with no significant adverse effects in the ecstasy group [66]. Other research on this drug is heavily confounded, as many ecstasy users are polydrug users, meaning they take other illicit drugs [81,82,83]. Studies that have attempted to identify simple dose–response effects have been futile [81,82,83]. Even though these substances have portrayed positive outcomes in their utilization to treat certain psychiatric illnesses, more observations are needed to confirm their safe therapeutic use.

The use of psychoceuticals in substance abuse disorders has also undergone clinical testing. There are mixed findings about ibogaine and noribogaine regarding their therapeutic usage in managing opioid craving and withdrawal symptoms [84,85,86]. These two drugs have shown a reduction in craving and withdrawal symptoms [87,88], yet other studies have found that they do not have significant effects on opioid withdrawal [89]. Clinical research on ibogaine is challenging in the United States due to its classification as a Schedule I substance [90]. While salvinorin A data for substance abuse treatment have not been found, the dose-related effects in healthy hallucinogen-using adults suggest this drug may be used for substance abuse conditions. A double-blind, placebo-controlled study demonstrated that the drug’s peak effects occurred two minutes after administration and rapidly dissipated [91]. The subjects were followed up with one month after the trial, and no persisting effects were observed [91]. Given that this drug produces both subjective and cognitive effects and some classic hallucinogen effects [91], its usage may be important in therapeutic settings.

NAC is reported to have positive clinical outcomes between the remaining three drugs, while peyote and ketamine’s therapeutic use must be further observed. First, NAC has shown potential in treating vascular and non-vascular neurological disorders, such as traumatic brain injury and cerebral ischemia [43]. Second, peyote has not elicited significant psychological or cognitive deficits in Navajo Native Americans [92]. These findings may not be generalizable to illicit hallucinogen users [92]. Third, the results and conclusions of a trial in China administering esketamine, a ketamine enantiomer, in patients undergoing major surgery, which evaluated outcomes such as remission rate three days post-operation and depression-related scores, were not reported in [93].

Overall, there are mixed findings on the therapeutic effects of the 11 drugs in the studies found within the search. Some of the drugs have shown positive treatment outcomes in animal- or human-model studies. In contrast, others have not demonstrated any significant clinical effects or have reported adverse effects. Therefore, more trials on these drugs’ safety and efficacy should be performed.

### 2.4. Limitations

This review does not represent an overall image of current research and development of psychoceuticals. It involves 20 drugs from six pharmaceutical organizations. Additionally, some of the drugs listed are still early in trial testing. Therefore, there are not much data on the therapeutic effects of these products, leading to the necessity of filling gaps in information using the organizations’ websites, which can lead to bias in their performance.

### 2.5. Future Directions

Creating a picture of the current psychoceutical availability in the global pharmaceutical market is a considerable next step. Evaluating the drugs’ clinical benefits and potential risks can illustrate if their positive outcomes outweigh their negative effects and, if they do, by how much. Additionally, it is essential to determine which psychoceutical drugs currently in the market may be replaced by drugs that are in clinical development. Finally, for those new prospective medications, it would be beneficial to compare trial results (effective concentration dose, clinical outcomes, adverse effects) between them and those already in the global market.

## 3. Methods

PRISMA statement registration was not obtained for this study but will be obtained for related studies in the future. Seven websites of psychoceutical organizations (Psycheceutical, GH Research, Atai Life Sciences, Cybin, Compass, MindMed, and Seelos Therapeutics) were utilized to evaluate the drugs they were researching or developing for clinical treatment of neurological and psychiatric illnesses. This review aimed to assess a list of 20 psychoceuticals. From those websites, a list of 19 drugs was composed to evaluate a combination of old and new engineered medications. One drug was obtained from the literature to refrain from restricting this study’s evaluation of newly engineered drugs. For psychoceutical assessment, the biochemical mechanisms, benefits and risks, and clinical trials were searched.

**Figure 2:**
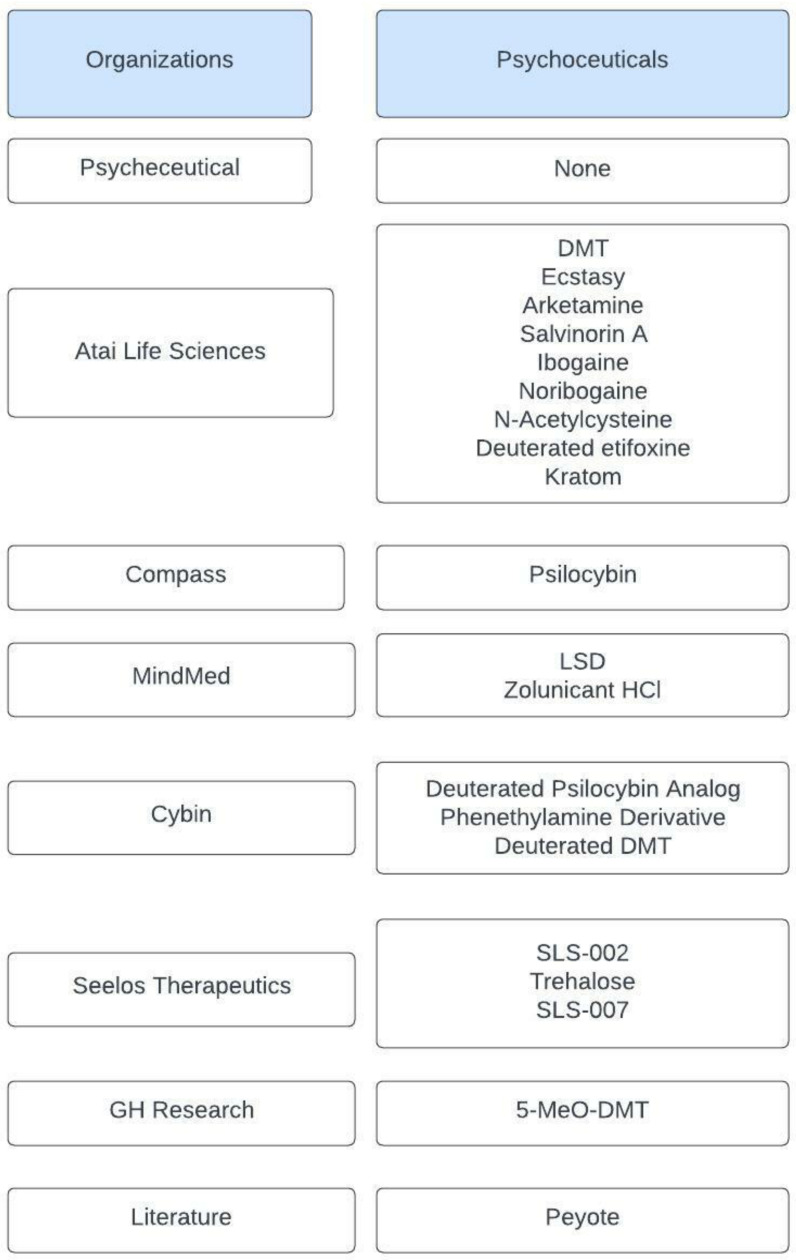
Psychoceuticals selected and their sources.

Out of the seven websites, only six presented psychoceuticals, while the organization that did not, Psycheceutical, only presented novel drug-delivery methods, which do not fall in line with the aim of this review. From GH Research and Compass, one drug was selected from each, 5-MeO-DMT (GH Research) and psilocybin (Compass). From three organizations, two to three drugs were selected: LSD and Zolunicant HCl (MindMed); Deuterated Psilocybin analog, Phenethylamine derivative, and Deuterated DMT (Cybin); SLS-002, Trehalose, and SLS-007 (Seelos Therapeutics). As a percentage of the drugs selected were psychedelics, such as LSD and psilocybin, an additional drug within that category was searched in PubMed, inputting the word psychedelic, and Peyote was chosen (Figure 2).

For biochemical mechanisms, the pathways or actions of these molecules were searched for in PubMed. In this search, phrases used included “drug name” and one of the following: biochemistry, mechanism, biochemical mechanism, and biochemical pathway. For the medications not found in the search or missing details, other sources on Google Scholar were used along with information provided by the organizations’ websites.

As if for the biochemical mechanisms, the benefits and risks of these twenty medications were searched for in PubMed. In the search, phrases included the “drug name” and one of the following: benefits, clinical benefits, risks, and potential risks. For the medications not found, information from sources found in Google Scholar search and the organization websites were used.

Only Pubmed, NIH clinical trial databases, and news reports for the clinical trial and trial review search were strictly used. Information from the organizations’ websites was excluded to avoid bias.

## 4. Conclusions

The 20 psychoceuticals presented in this review have shown pre-clinical or clinical promise. Many of these drugs, however, are far from entering the market. Although the organizations publish an overview of their results on their websites, finished clinical trial studies in the past literature and from the NIH clinical trial database for numerous drugs in the list were not found. The targets of these drugs have been identified, but some underlying biochemical mechanisms still need to be confirmed or researched thoroughly before conclusions may be made. Additionally, more than half of the drugs on the list have side effects that are important to consider for future patient treatment use. Still, they may improve the treatments of multiple illnesses and conditions, ranging from neurologic, such as TBI, to psychiatric, such as substance abuse disorder. The biochemical mechanisms, benefits, and risks were not all found in the literature search. Information from the organizations’ websites had to be considered, as many of these are newly engineered.

The application of these drugs within neuro-related specialties, including neurology and neurosurgery, requires further exploration and research. It is crucial to recognize that only a small subset of psychoceuticals was reviewed. It is worth reviewing the applications of other drugs within neuro-related and psychiatric fields.

## Figures and Tables

**Table 1:** Biochemical structures, mechanisms, and receptor functions of twenty psychoceutical drugs.

Drug Name	Chemical Structure	Target Type	Method of Action	Citations
**5-Methoxy-N,N-dimenthyltryptamine** **(5-MeO-DMT)**	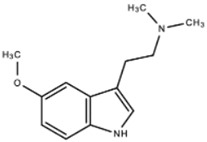	Serotonin Receptor	5-MeO-DMT acts as a 5-HT analog in the serotonin system with a high affinity for the 5-HT_1A_ receptor.	Shen et al. [9] Yu [10]
**N,N-dimethyltryptamine (DMT)**	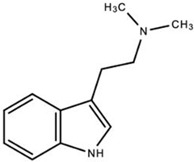		DMT activates pharmacological receptors, such as serotonin receptors, within the synaptic cleft after its secretion as a neurotransmitter. Although the exact molecular pathway of DMT remains unclear, the effects of DMT as a modulator for serotonergic systems are currently being tested.	Carbonaro, Gatch [11]
**MDMA-Derivative (Ecstasy)**	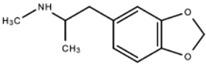		MDMA and derivatives increase serotonin and norepinephrine release while blocking their reuptake within the synaptic cleft.	National Institute on Drug Abuse [12]
**Lysergic Acid Diethylamide** **(LSD)**	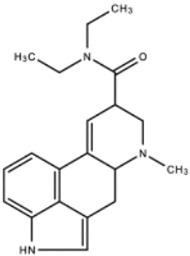		LSD functions to increase serotonin concentration and acts as a 5-HT_1A_ and 5-HT_2_ receptor agonist. LSD has inhibitory effects acting upon 5-HT_1A_ receptors and stimulating effects acting upon 5-HT_2_ receptors.	Andén et al. [13] Passie et al. [14]
**Peyote**	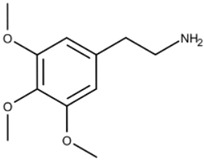 * Mescaline, the active component of Peyote		Mescaline, the active component of Peyote, functions as a serotonin receptor agonist, especially 5-HT_2_ receptors, with a higher affinity for 5-HT_2C_ receptors than for 5-HT_2A_ receptors and 5-HT_2B_ receptors. Peyote increases cytosolic levels of IP3 through Gq coupling of G-proteins to activate phospholipase C, stimulating calcium channel opening.	Dinis-Oliveira et al. [15]
**Deuterated Psilocybin Analog** **(CYB003)**	*		Once converted to psilocin, CYB003 functions as a partial agonist of 5-HT_2A_ receptors, a subtype of 5-HT_2_ receptors that act as serotonin and G protein-coupled (GCP) receptors.	Cybin [16] Schmidt et al. [17]
**Deuterated DMT** **(CYB004)**	*		CYB004 acts as an agonist of 5-HT_2A_ serotonin receptors. Uptake of DMT occurs through serotonin uptake transporters (SERT) and is sequestered into synaptic vesicles via monoamine transporters.	Cybin [18] Barker [19] Carbonaro, Gatch [11]
**Phenethylamine derivative** **(CYB005)**	*		CYB005 inhibits serotonin and dopamine transporters within the synaptic cleft.	Cybin [20] RxList [21] Irsfeld et al. [22]
**Psilocybin** **(COMP360)**	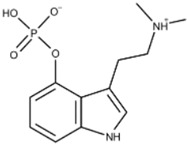		COMP360 acts as an agonist of 5-HT_2A_ receptors, a subtype of 5-HT_2_ receptors that act on serotonin and G protein-coupled (GCP) receptors.	Compass [23] Schmidt et al. [17]
**(R)-Ketamine (Arketamine)**	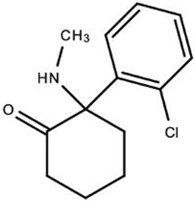	NMDA Receptor	Arketamine acts as an NMDA receptor inhibitor with long lasting effects. Although the metabolism of Arketamine to 2R,6Rhydroxynorketamine (HNK) is known to have antidepressant effects, the exact molecular mechanisms of arketamine remain unclear.	Zanos et al. [24] Wei et al. [25]
**SLS-002 (Ketamine)**	*		SLS-002 functions as an NMDAr antagonist that binds to GABAergic interneurons, resulting in the disinhibition of glutamatergic neurons. A glutamate surge follows, stimulating AMPA receptors to potentiate BDNF and mTORC1 signaling pathways.	Matveychuk et al. [26] Derakhshanian et al. [27]
**Deu-mitragynine (Kratom)**	*	Opioid Receptor	Deu-mitragynine acts as a mu-opioid (MOR) agonist with the ability to interact with G-protein coupled receptors, resulting in its analgesic effects. Deu-mitragynine has been shown to exhibit opioid-receptor-dependent analgesic effects and G-protein-based agonists of MOR.	Atai Life Sciences [28] Shukla et al. [29]
**Salvinorin A**	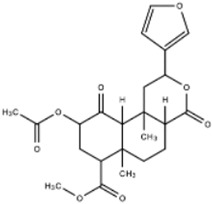		Salvinorin A acts as an agonist of k-opioid receptors (KOPr) and is coupled to Gi/o proteins. Salvinorin A inhibits adenylyl cyclase and activates beta-arrestin mediated pathways and ERK/MAPK pathways.	Brito-da-Costa et al. [30] Roach, Shenvi [31]
**12-methoxyibogamine** **(Ibogaine)**	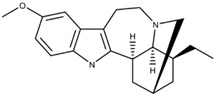	Serotonin Receptor NMDA Receptor Opioid Receptor	Ibogaine functions as a kappa opioid receptor agonist, NMDA receptor antagonist, serotonin uptake receptor antagonist, and nicotinic receptor antagonist. Ibogaine’s underlying mechanism remains unclear.	Glick et al. [32] Healthtown [33] Atai Life Sciences [34]
**12-hydroxyibogamine** **(Noribogaine)**	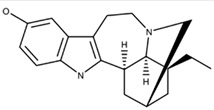		Noribogaine is the main metabolite of Ibogaine and has a higher affinity for opioid receptors than its parent compound. Noribogaine acts as an NMDA receptor antagonist, k-opioid receptor agonist, serotonin reuptake inhibitor, and µ-opioid receptor antagonist. The exact mechanism behind its effects on k-opioid receptors remains unclear.	Maillet et al. [35] Atai Life Sciences [34]
**Trehalose** **(SLS-005)**	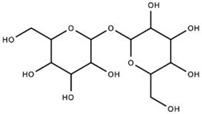	GLUT8 Receptor	Trehalose stabilizes proteins, enhances autophagy, and enhances lysosomal pathways through mTOR-independent pathways. By increasing cytosolic levels of light chain 3-II, the inner and outer membranes of autophagosomes prepare for lysosome fusion. SLS-005 must act upon the mammalian trehalose transporter (GLUT8) for trehalose-induced autophagy in hepatocytes, but is unknown in neuronal cells.	Seelos Therapeutics [36] Rusmini et al. [37] Lee et al. [38] Sarkar et al. [39]
**SLS-007**	*	Peptide	SLS-007 inhibits protein aggregation by targeting alpha-synuclein’s non-amyloid component cores (NAcore).	Inc ST [40] Seelos Therapeutics [41]
**N-acetylcysteine (NAC)**	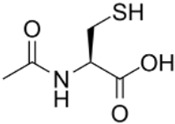	NF-κB	NAC increases intracellular glutathione (GSH) concentration, modulates glutamatergic, neurotrophic, and inflammatory pathways, and suppresses NF-κB activity.	Tenório et al. [42] Bavarsad [43]
**Zolunicant HCL** **(MM-110)**	*	α3β4 receptors	MM-110 functions as an inhibitor for nicotinic α3β4 receptors and regulates dopamine levels during withdrawal.	O’Brien [44]
**Deuterated Etifoxine** **(Etifoxine-d3)**	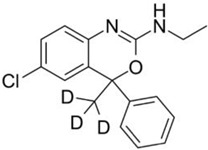	GABA receptors	Deuterated etifoxine targets GABA channel subunits and projects GABAergic transmission via GABA receptors. Deuterated etifoxine can increase mitochondrial translocator protein (TSPO) to stimulate neurosteroid synthesis.	Atai Life Sciences [45]

Biochemical structures, mechanisms, and receptor functions of twenty psychoceutical drugs. The table includes the chemical structures of known drugs, their receptor target type, and their biochemical method of action. Unknown chemical structures are marked with an asterisk (*).

**Table 2:** Highlights of the psychoceutical benefits.

Drug	Benefit	Citations	Drug	Benefit	Citations
LSD	Treats chronic alcoholism and anxiety	Bodnár, Kakuk [52]	MM-110 (zolunicant HCl)	Potential clinical utility to safely mitigate symptoms of opioid withdrawal	O’Brien [44]
5-methoxy-N, N-dimethyltryptamine (5-MeO-DMT)	Fewer suicidal thoughts, plans, attempts, and psychological distress	Lancelota, Davis [53] Davis et al. [54]	CYB005 (Phenethylamine derivative)	Treats neuroinflammation and psychiatric conditions	Cybin [20] Cybin [55]
Ibogaine	Humans: reduces cravings for heroin and withdrawal symptoms Rats: reduces opioid withdrawal, as well as cocaine and heroin self-administration	Corkery [46]	CYB004 (Deuterated DMT)	Anxiety disorders, including generalized anxiety disorder, GAD, and social anxiety disorder, SAD.	Cybin [18]
Noribogaine	Blocks opioid cravings Mice: fewer stress effects and less acute toxicity	Glue et al. [56] Mash [57]	CYB003 (Deuterated Psilocybin Analog)	Treats major depressive disorder (MDD) and alcohol use disorder (AUD).	Cybin [16]
Peyote (mescaline)	Improves depression, anxiety, and PTSD	Uthaug et al. [58]	Deuterated etifoxine	Treatment of anxiety	Golani et al. [59] Witkin et al. [60]
Salvinorin A	Feelings of calm and relaxation	Listos et al. [61] Brito et al. [30]	SLS-005 (Trehalose)	Improving functional outcomes following TBI	Portbury et al. [62]
Arketamine	Antidepressant	Zanos et al. [24] Leal et al. [63]	Psilocybin (COMP360)	Psilocybin significantly reduces depressive symptoms	Johannesdottir, Sigurdsson [64]
Ecstasy	Mice: extinction of fear memories Humans: attenuated amygdala response when shown negative stimuli	Holland [65] Mitchell et al. [66]	Ketamine	Anti-depressant, pain management, anti-inflammatory	Gao et al. [67]
N-acetylcysteine	Improves cognition in schizophrenics	Schwalfenberg [68] Muhammed, Vearrier [69]	SLS 007	Treatment for PD	Inc ST [40] Vidović, Rikalovic [70]
N,N-dimethyltryptamine	Anti-anxiety/anti-psychotic via actions at the trace amino acid receptor	Carbonaro, Gatch [11]	Kratom	Substitute for opioids among people who are addicted. Kratom also enhances mood and relieves anxiety	Swogger, Walsh [71]

## Data Availability

No new data were created or analyzed in this study. Data sharing is not applicable to this manuscript.
